# Supplementary material for: Molecular and spatial epidemiology of HCV among people who inject drugs in Boston, Massachusetts
Source: PLoS One. 2022 Aug 25;17(8):e0266216. doi: 10.1371/journal.pone.0266216 (PMC9409531; doi:10.1371/journal.pone.0266216)
Supplement: S1 Table — (DOCX) [file pone.0266216.s001.docx]

**Supplemental Table**

Supplemental Table. HCV Subtype by town of first injection and number of years injecting, Boston and Cambridge, Massachusetts, 2016.

| HCV  Subtype | Town Where First Injected | | | |
| --- | --- | --- | --- | --- |
|  | In-Boston | In Suburbs  (On T-Line) | Beyond the Suburbs  (>T-Line) | Missing |
| Subtype 1a | 11 | 7 | 16 | 3 |
| Subtype Non-1a | 9 | 7 | 7 | 4 |
|  |  |  |  |  |
| HCV  Subtype | Number of Years Injecting | | | |
|  | 0-5 years | 6-10 years | >10 years | Missing |
| Subtype 1a | 7 | 10 | 15 | 4 |
| Subtype Non-1a | 3 | 3 | 16 | 4 |
