# Supplementary material for: Molecular and spatial epidemiology of HCV among people who inject drugs in Boston, Massachusetts
Source: PLoS One. 2022 Aug 25;17(8):e0266216. doi: 10.1371/journal.pone.0266216 (PMC9409531; doi:10.1371/journal.pone.0266216)
Supplement: S1 File — (PDF) [file pone.0266216.s002.pdf]

Subject: FW: genbank
Date: Friday, June 17, 2022 at 3:20:12 PM Eastern Daylight Time
From: Paintsil, Elijah
To: Heimer, Robert

Here're the accession numbers of sequences:

BankIt2593684 PUGG41782 ON782019
BankIt2593269 VLOB091677 ON758698
BankIt2593269 CUSE030887 ON758699
BankIt2593269 JSML081386 ON758700
BankIt2593269 BATL082395 ON758701
BankIt2593269 CUMR041691 ON758702
BankIt2593269 CRPN091783 ON758703
BankIt2593269 JSWB090186 ON758704
BankIt2593269 DNMR041285 ON758705
BankIt2593269 TALV022388 ON758706
BankIt2593269 JSRA090383 ON758707
BankIt2593269 CTRC081269 ON758708
BankIt2593269 JSCN1000783 ON758709
BankIt2593269 LNWN051472 ON758710
BankIt2593269 SASI041686 ON758711
BankIt2593269 JMBR042686 ON758712
BankIt2593269 JMRA101871 ON758713
BankIt2593269 MHKL110987 ON758714
BankIt2593269 DVYU042187 ON758715
BankIt2593269 JSBY123072 ON758716
BankIt2593269 JSCV081884 ON758717
BankIt2593269 RMMT011580 ON758718
BankIt2593269 JRJN090377 ON758719
BankIt2593269 CASE020887 ON758720
BankIt2593269 JNKR11687 ON758721
BankIt2593269 ENSNO71477 ON758722
BankIt2593269 TNJH070879 ON758723
BankIt2593269 JMCL102377 ON758724
BankIt2593269 FEMN010881 ON758725
BankIt2593269 KRFN031571 ON758726
BankIt2593269 AACS030887 ON758727
BankIt2593269 HCCR062271 ON758728
BankIt2593269 JSMC031185 ON758729
BankIt2593269 MTWI110491 ON758730
BankIt2593269 TRRE80686 ON758731
BankIt2593269 VLDE0511191 ON758732
BankIt2593269 DNRG041191 ON758733
BankIt2593269 EESH120474 ON758734
BankIt2593269 AABA88852 ON758735
BankIt2593269 JNFO091286 ON758736
BankIt2593269 CRAU052990 ON758737
BankIt2593269 SMSC122679 ON758738

BankIt2593269	BEAS050984	ON758739
BankIt2593269	JSSE061388	ON758740
BankIt2593269	EMSE093081	ON758741
BankIt2593269	NSGB080785	ON758742
BankIt2593269	DVHR110288	ON758743
BankIt2593269	FAMD0432776	ON758744
BankIt2593269	AGDM123184	ON758745
BankIt2593269	JHAA081283	ON758746
BankIt2593269	JMMC042291	ON758747
BankIt2593269	SEBO081387	ON758748
BankIt2593269	JSNL72290	ON758749
BankIt2593269	AGCZ061385	ON758750
BankIt2593269	ADBA123175	ON758751
BankIt2593269	DRSN041585	ON758752
BankIt2593269	DRCA070885	ON758753
BankIt2593269	MCMO051082	ON758754
BankIt2593269	JNBO121483	ON758755
BankIt2593269	JNCM070486	ON758756
BankIt2593269	MCSL031487	ON758757
BankIt2593269	CRND	ON758758

Lei Zhou

Postdoctoral Associate

Department of Pediatrics, Infectious Diseases

Yale School of Medicine
