# Supplementary material for: Molecular and spatial epidemiology of HCV among people who inject drugs in Boston, Massachusetts
Source: PLoS One. 2022 Aug 25;17(8):e0266216. doi: 10.1371/journal.pone.0266216 (PMC9409531; doi:10.1371/journal.pone.0266216)
Supplement: Attachment — Submitted filename: Responses to Reviewer_July revision.docx [file pone.0266216.s004.docx]

**Responses to Reviewer #2**

*Reviewer #2: I am happy with the authors responses to my comments. After reviewing the updates I have a couple more minor queries relating to Figure 1B –*

*I note that genotype 1b sequences cluster with gt2/4 sequences, rather than with gt1a sequences – I assume this is because core is being used rather than NS5B or whole genome? It might be worth commenting on this in the figure legend for those less familiar with HCV phylogenies if so.*

We have added this point to the figure legend, which now reads: “

*Could the authors add more information on what exactly the labels on the tree mean into the figure legend? I initially assumed they were labelling HCV subtypes, but I don’t think this is correct. If they are highlighting more closely related sequences, could they please list the criteria (ie distance threshold) for doing this?*

We address this point in the revision by amending the text to read: “The double-headed arrows indicate connection by referral (n=31). This provides visual evidence that the social network that was accessed to recruit participants contained people infected with different strains of HCV.”
